# Recent Advances in Cancer Stem Cell-Targeted Immunotherapy

**DOI:** 10.3390/cancers11030310

**Published:** 2019-03-05

**Authors:** Narayanasamy Badrinath, So Young Yoo

**Affiliations:** 1 Biomedical Sciences, School of Medicine, Pusan National University, Yangsan 50612, Korea; badrisamy@gmail.com; 2 Research Institute for Convergence of Biomedical Science and Technology, Pusan National University Yangsan Hospital, Yangsan 50612, Korea; 3 BIO-IT Foundry Technology Institute, Pusan National University, Busan 46241, Korea

**Keywords:** cancer stem cells, immunotherapy, combination therapy

## Abstract

Cancer stem cells (CSCs) are one of the reasons for the relapse of cancer cells and metastasis. They have drug resistance against most chemotherapeutic agents. CSCs are also responsible for tumor cell heterogeneity and cause minimal residual disease. In order to achieve complete regression of tumors, CSCs have to be targeted. Recent advances in immunotherapies have shown promising outcomes in curing cancer, which are also applicable to target CSCs. CSCs express immune markers and exhibit specific immune characteristics in various cancers, which can be used in immunotherapies to target CSCs in the tumor microenvironment. Recently, various strategies have been used to target CSCs. Adaptive T-cells, dendritic cell (DC)-based vaccines, oncolytic viruses, immune checkpoint inhibitors, and combination therapies are now being used to target CSCs. Here, we discuss the feasibility of these immunological approaches and the recent trends in immunotherapies to target CSCs.

## 1. Introduction

According to cancer stem cells (CSCs) theory, CSCs are small numbers of cells that are hidden in tumors and fuel cancer growth [1]. CSCs have the capacity for self-renewal, differentiation, and tumorigenicity if relocated into an animal model [2]. The existence of CSCs or cancer-initiating cells has been reported in various cancers [3,4,5,6]. One of the greatest therapeutic struggles with cancer is to eradicate CSCs [7]. The relapse of cancer cells, heterogeneity of tumor cells, metastasis, and minimal residual disease are the major consequences of CSCs [8]. CSCs are resistant to conventional therapies, and escaped CSCs keep inducing tumor formation even after complete eradication of adult cancer cells [9]. Epithelial mesenchymal transition (EMT), interleukin-4 (IL-4) signaling, drug efflux proteins, and upregulation of aldehyde dehydrogenase (ALDH) activity are perhaps the reasons for the resistance of CSCs to conventional therapies [10]. The aberrant expression of Janus-activated kinase/signal transducer and activator of transcription, Hedgehog, Wnt, Notch, phosphatidylinositol 3-kinase/phosphatase and tensin homolog, and nuclear factor-κB signaling pathways in various CSCs have been reported [5]. In order to distinguish them from just cancer cells, different markers have been used. Most of the studies reported that the main CSC markers are CD133, CD44, IL-6R, and ALDH [11]. The CSC niche of the tumor microenvironment (TME) plays important roles in the metastasis of cancer cells, which has been reported in various cancer models [12]. Endothelial cells, myofibroblasts, and pericytes in niche participate angiocrine signals, malignant conversion, and the protection of metastasis, respectively. Co-inhibitory molecules and immune checkpoint ligands, such as programmed death-ligand 1 (PD-L1) and programmed death-ligand 2 (PD-L2), are highly expressed on CSCs of various cancers. PD-1 is receptor for these ligands, which express on immune cells. The interaction between PD-L1/PD-L2 and PD-1 aids CSCs in escaping from the killing [13,14]. In order to target these molecules of CSCs, the immune checkpoint blockade of anti-PD-L1 has been used. Previously published review articles elaborate strategies of targeting CSCs using these markers, but the major limitation is paucity of immune molecules targeting [11,15,16].

In this review, in order to understand immunotherapy-based targeting of CSCs, we covered topics related to CSCs and stem cells, surface receptors, immune escaping mechanisms, and recent trends in CSC-targeted immunotherapy.

## 2. CSCs and Normal Stem Cells

Normal stem cells and CSCs have similar functional capabilities. Both cells can proliferate extensively with a self-renewal ability [17]. In order to identify CSC populations in solid tumors, specific surface markers are used. Despite the fact that normal stem cells and CSCs share most markers (CD29, CD44, CD133, etc.) [18], the coexpressions of CD176 (Thomsen-Friedenreich antigen) and other surface markers can be used to characterize CSCs in tumors. Populations of CD44^+^_,_ CD133^+^, CD176^+^ CSCs were reported in lung, breast, and liver cancers [19]. In prostate cancer, coexpressions of CD44, α2β1 integrin, CD133, CD49f, and CD176 were characterized as stem cell-like cells [20].

Mutations in stem cells can raise cancer stem-like cells, and some studies reported this transformation. Genomic instability and abrogated tumor suppression mechanisms are associated with this transformation [21]. Environmental aberrancy during differentiation of embryonic stem cells leads to CSCs, which are characterized by spontaneously accumulated DNA lesions with senescence and apoptosis resistance [22]. Malignant liposarcomas were aroused from induced pluripotent stem cells under the influence of tumor-derived extracellular vesicles, which were isolated from the conditioned medium of a mouse lewis lung carcinoma cell line [23]. The oncogenic manipulation of mouse embryonic stem cells can generate cancer-like stem cells, which was reported in an ovarian teratoma in vivo model. The insertion of oncogenic elements—SV40 LTg and HrasV12—by using a mouse stem virus long terminal repeat-based retroviral system induced cancer-like stem cells [24].

The formation of CSCs from nonstem cancer cells (NSCCs) has also been reported. Interleukin-6 mediates the maintenance of tumor heterogeneity through a dynamic equilibrium between CSCs and NSCCs. The conversion of NSCCs to CSCs was reported in genetically different breast cell lines, human breast tumors, and a prostate cell line. This transformation is mediated by IL-6 secretion. Differential expressions of various microRNAs were also reported in this transformation [25]. The role of hypoxia in CSCs formation from NSCCs was demonstrated in colorectal cell lines. Hypoxia prevents differentiation of enterocytes and goblet cells by downregulating CDX1 and Notch1 [26].

## 3. Surface Receptors on CSCs

CSCs express various immune receptors on their surfaces. These receptors play key roles in the therapeutic resistance and metastasis of cancers. The roles of CSC surface receptors in tumorigenesis and immune resistance have been reported. The leucine-rich repeat-containing G-protein-coupled receptor 5 (Lgr5) is identified as colorectal cancer (CRC). CSCs and its cell ablation restricts primary tumors, but they do not completely suppress tumor formation. Proliferative Lgr5^−^ cells attempt to replenish the Lgr5^+^ CSC pool in the TME and promote rapid re-initiation of tumor growth upon treatment cessation [27]. CD95 expression and CD95 signaling are associated with EMT differentiation programs in gastrointestinal cancer [28]. It is also demonstrated that stimulation of CD95 maintains the CSC pool of an increased number of cancer cells with stem cell traits [29]. The constitutive expression of HLA-E on glioblastoma stem-like cells inhibits NK cell-mediated lysis [30]. CD133^+^ CSCs in colon cancer are resistant to apoptosis due to production of IL-4. Treatment with an IL-4Rα antagonist or anti-IL-4 neutralizing antibody enhanced the antitumor efficacy of standard drugs and confirmed the autocrine mechanism of IL-4 in CSCs in colon cancer [31]. In CRC patients, the higher CD133^+^ CSCs proportion was associated with lower numbers of activated dendritic cells (DCs) [32]. The expression pattern of three surface receptors—CD133, Trop-2, and α2β1 integrin—have been identified as putative markers in human prostate cancer [33]. Platelet-derived growth factor receptors α and β (PDGFR-α/β) were upregulated and promoted migration, invasion, and chemotherapy resistance in sarcoma CSCs. The PDGFR-α/β can be targeted as potential therapeutic candidates for sarcoma treatment [34]. In ovarian CSCs, receptor tyrosine kinase-like orphan receptor 1 (ROR1) expression revealed its functional role in promoting migration/invasion. Humanized mAb (specific for ROR1 (UC-961)) inhibited the capacity of ovarian cancer cells to migrate and form spheroides [35]. Toll-like receptor 4 expression on CSCs of hepatocellular carcinoma (HCC) was reported. It was associated with tumor invasion, migration, and a poor prognosis of HCC [36]. The high expression of MHC I in melanoma, colon cancer, and pancreatic cancer is associated with CDK1 upregulation. Further, the interaction between CDK1 and Sox2 promotes tumor initiation in human melanoma [37]. Inhibition of the MDM2-p53 interaction reduces ALDH^high^ and CD44^high^ CSCs in mucoepidermoid carcinoma. A marked decrease in expression of Bmi-1 and in a fraction of ALDH^high^ CD44^high^ was demonstrated in this model [38]. Rapid tumorigenesis was associated with the surface expression of PD-L1, E-cadherin, CD24, and VEGFR2 in epithelial CSCs, which was established from the ascites of a bladder cancer patient. These surface expressions were also closely linked with signatures of immune evasion, increased stemness, increased calcium signaling, transformation, and novel E-cadherin–RalBP1 interaction [39]. PD-L1 expression on CSCs of breast cancer promotes the expression of OCT-4A and Nanog transcription factors. These expressions sustain the stemness of breast cancer through the activation of the PI3K/AKT pathway [40]. Apart from these roles, some of the surface receptors have been commonly used to characterize CSCs and projected as biomarkers in various cancers (Table 1).

## 4. Immune Escaping of CSCs and TME

**Figure 1:**
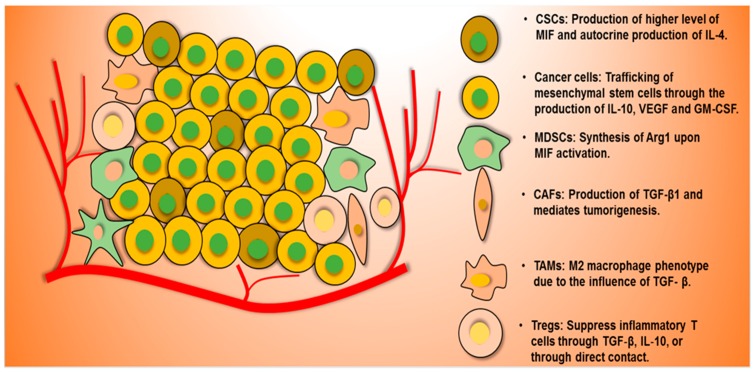
General roles of CSCs and other cells in tumor microenvironment (TME) and the mechanisms of immune escaping, and tumorigenesis. CSCs produces of higher level of migration inhibitory factor (MIF) and autocrine production of IL-4 in order to escape macrophage killing. Cancer cells produce IL-10, VEGF, and GM-CSF, which are involved in trafficking of mesenchymal stem cells. Myeloid derived suppressor cells (MDSCs) secrete Arg1 through MIF1 activation. CAFs mediate tumorigenesis through TGF-β. M2 macrophage promotes tumorigenesis for TGF-β response. TGF-β and IL-10 of T-regulatory cells suppress T-effector cells. (Abbreviations: CAFs, cancer-associated fibroblasts; CSC, cancer stem cells; FAS, fas cell surface death receptor; GM-CSF, granulocyte-macrophage colony-stimulating factor; IL-10, interleukin-10; MDSCs, myeloid-derived suppressor cells, TGF-β, tumor growth factor-beta; VEGF, vascular endothelial growth factor).

CSCs, having a low expression of MHC I molecules, natural killer cells (NK cells) receptors, and other innate immune receptors, can escape from killing by cancer killer cells such as NK cells and T-cells. Most of the signaling pathways, which participate in normal stem cell physiological roles, such as EGF/EGFR, FGF/FGFR, Hedgehog, HER2, JAK/STAT, MAPK, Myc, NF-κB, PTEN/PI3K, and Wnt, are dysregulated in CSCs as receptors, ligands, oncogenes, or transcription factors, according to the context and types of cancers. It has also been observed that normal stem cells markers (Nanog, Sox2, Oct4, and Klf) are over-expressed in CSCs. These aberrant expressions make CSCs immunoresistant against antitumor immunity [49,50]. In addition, a TME consists of both immune cells and stromal cells, with cancer cells as well as CSCs; tumor-associated macrophages (TAMs), tumor-infiltrating lymphocytes (TILs), regulatory T-cells, myeloid derived suppressor cells (MDSCs), DCs, NK cells, and natural killer T-cells are filtrated from bone marrow. Stromal cells include blood and lymphatic endothelial cells and cancer-associated fibroblasts [51]. They secrete various cytokines and chemokines (which are also responsible for their antitumor immunity), metastasis, and suppression in the anticancer therapeutic efficacy [52,53]. The interactions between CSCs and cellular components through these cytokines and chemokines severely suppress antitumor immunity [54]. The general roles of each type of cells in a TME are shown in Figure 1. For instance, the markers expressed in CSCs and immune cells affect the prognosis of stage III colon cancer patients. Patients categorized as SOX2^low^/FoxP3^high^ expression showed good prognosis, whereas patients categorized as SOX2^high^/PD-L1^low^ indicated poor prognosis [55]. Furthermore, other mechanisms have been reported in various cancers and are summarized in Table 2.

## 5. Targeting CSCs by Immunotherapy

Immunotherapy targets CSCs through immune cells such as cytokine-induced killer (CIK) cells, NK cells, γδ T-cells, and CD8^+^ T-cells. DC-based vaccines also target CSCs [69]. Besides, oncolytic virotherapy (OVT) induces antitumor immunity through immunogenic cell death and the activation of the T-cell [70]. It also targets CSCs in combination with other immunotherapies. Recently, most of the targeting strategies use combination therapy, which is commonly consisted of DC-based vaccines, oncolytic viruses, and immune checkpoint blockades. The eradication of CSCs and its therapeutic efficacy are usually associated with the infiltration of lymphocytes, M1 macrophage polarization, and the induction of antitumor cytokines in the TME. The targeting strategies against CSCs in various cancers are listed in Table 3.

### 5.1. Adoptive T-Cell Therapy

Adoptive T-cell therapy (ACT) is a type of personalized therapy that uses cancer-bearing host immune cells with direct anticancer activity to treat cancer. TILs from naturally occurring tumors have intrinsic antitumor activity. ACT involves the isolation of TILs from a patient, cultured in the presence of IL-2 and evaluation of specific tumor recognition. These processes are followed by the reinfusion of selected TILs to the same patient [71]. Recently, engineered T-cells with chimeric antigen T-cell receptors (CAR T-cells) against the antigens of CSCs have also been developed and evaluated in various cancer models. In prostate cancer, CAR T-cells targeted against EpCAM antigens eradicated CSCs in PC3M and PC3 tumor models [72]. CAR T-cells engineered with membrane-bound chimeric IL-15 induce CSC memory T-cells in tumor-specific T-cells in CD19^+^ leukemia [73]. The selective killing of CSCs by adoptively transferred CD8^+^ cytotoxic T-cells, specific for the CSCs antigen ASB4, was reported colon cancer [74].

Adoptive immunotherapy also includes the adoptive transfer of cytokine-induced killer (CIK) cells. CIK cells with CAR T-cells that are transduced against CD123 strongly killed CD123^+^ cell lines and primary acute myeloid leukemia cells [75]. MHC-independent antitumor activity in chemotherapy and BRAF inhibitor-surviving CSCs was reported in melanoma [76]. A partial NKG2D-ligands recognition with CSCs and its therapeutic efficacy by CIK cells was shown in HCC and nasopharyngeal carcinoma [77,78].

The adoptive transfer of NK cells from healthy donors showed the killing of stem-like and differentiated tumor cells upon activation with IL-2 and IL-15. CSCs that shifted towards being susceptible to cisplatin therapy were also noted in high-grade non-muscle invasive bladder cancer [79]. In various cancer models, the adoptive transfer of NK cells led to upregulation of NK cells activation ligands, such as MICA/B, Fas and DR5 on CSCs. NKG2D-dependent mechanisms of killing of CSCs were also revealed in these models [80]. The adoptive transfer of NK cells in oral squamous carcinoma showed the expansion and functional activation of super-charged NK cells by osteoclast in both an IL-12- and an IL-15-dependent manner against CSCs [81].

The adoptive transfers of γδ and CD8^+^ T-cells upregulated MHC class I and CD54/ICAM-1 on CSC-like cells and induced antigen specific-killing by CD8^+^ T-cells in breast cancer. Synergism between MHC-restricted and non-MHC-restricted T-cells was shown in this model [82].

### 5.2. DC-Based Vaccines

The therapeutic efficacy of DC-based vaccines against CSCs have been reported in various cancers. DCs pulsed with cancer cell lines or CSC lysates were used as vaccines to evaluate the therapeutic efficacy. In the malignant melanoma model, CSCs lysate-pulsed DCs induced IFN-γ and IL-4 secretion in vaccinated mice. These effects mediated the suppression of tumor growth and prolonged survival in immunized mice [83]. The downregulation of chemokine (C-C motif) receptors CCR7, CCR10, and their ligands CCL21, CCL27, and CCL28 were associated with therapeutic efficacy in melanoma and squamous cell carcinoma [84]. DCs charged with total lysates of Panc-1 CSCs induced INF-γ and IL-2 secretion, and mediated lymphocytes were reported in pancreatic cancer in an in vitro model [85]. DCs loaded with NANOG peptides induced highly specific anti-tumor T-cell responses against CSCs in ovarian cancer [86]. An ALDH^high^ SCC7-specific CSC-DC vaccine showed the reduction of local tumor relapse and prolonged host survival in squamous cell cancer. As a metastatic model, in D5 melanoma, the inhibition of primary tumor growth, reduced spontaneous lung metastases, and increased host survival were reported. These therapeutic efficacies were associated with the downregulation of CCR10 on ALDH^high^ D5 CSCs and its ligands on lung tissues [87]. Furthermore, the therapeutic efficacy of DC-based vaccines was successfully shown in immunocompetent murine models. Using ALDH^high^ CSC-pulsed DCs in D5 melanoma and SCC7 squamous cell cancer models, high levels of IgG bound CSCs and CSCs lysis in presence of complement were reported Cytotoxic T Lymphocytes (CTLs) harvested from peripheral blood mononuclear cells or splenocytes of vaccinated mice were also capable of killing CSCs in vitro [88].

### 5.3. Oncolytic Virotherapy (OVT)

OVT induces antitumor immunity through immunogenic cell death and the activation of T-cells. Various studies indicated the therapeutic efficacy of OVT against CSCs. Oncolytic herpes simplex virus armed with IL-12 (G47∆-mIL12) infection and the induction of tumor regression were reported in syngeneic mice bearing intracerebral 005 tumors. An IFN-γ release, the inhibition of angiogenesis, and a reduction of the number of regulatory T-cells in the tumor were also noted in glioblastoma [89]. The selective infection of CD133-targeted oncolytic adenovirus in CD133^+^ CSCs was also reported in glioblastoma [90]. The oncolytic vaccinia virus, GLV-1h68 strain selectively replication, and killing of stem cell-like cancer cells (higher ALDH1 activity) were reported in breast cancer model [91]. In ovarian cancer, the killing of CD44^+^ CD117^+^ cancer-initiating cells by CXCR4 antagonist expressed-oncolytic vaccinia virus infection was reported [92]. The cancer-favoring oncolytic vaccinia virus’ selective infection and therapeutic efficacy against stem-cell-like colon (CD133^+^ and CD44^+^) cancer cells in combine with fluorouracil were reported in colon cancer [42]. Oncolytic measles viruses targeted and lysed CD133^+^ tumor-initiating cells in HCC [93].

### 5.4. Other Immunotherapeutic Approaches

Other immunotherapeutic approaches, like blockades against immune receptors and ligands, were also used target CSCs in bulk tumors. A monoclonal antibody against Lgr5 in colon cancer showed the suppression of Lgr5, Wnt pathway in CSCs and tumor volume reduction [94]. In triple negative breast cancer, the IFN-β mediated suppression of E-M/CSC plasticity by re-engaging type I IFN signaling in CSCs was reported [49]. A blockade of the IL-8 receptor CXCR1 caused the induction of aggressive apoptosis through FASL/FAS signaling and it was mediated by the FAK/AKT/FOXO3A pathway in CSCs of breast cancer [95].

### 5.5. Combination Immunotherapy

To target and complete eradicate of CSCs, combined immunotherapy approaches have been developed recently. A DC-based vaccine in combination with anti-PD-L1 and anti-CTLA-4 showed the elimination of ALDH^high^ CSCs, enhanced T-cell expansion, suppressed TGF-β secretion, enhanced IFN-γ secretion, and significantly enhanced host specific CD8^+^ T-cells against CSCs in mouse melanoma [96]. An oncolytic herpes simplex virus expressing IL-12 eradicated glioblastoma stem-like cells in combination with anti-PD-1 and anti-CTLA-4. CD4^+^, CD8^+^ T-cells, intratumoral M1-like macrophages, and an increased ratio of ‘T effector: T regulatory cells’ was responsible for the therapeutic efficacy of triple combination efficacy in glioblastoma [97]. In another glioblastoma study, STDENVANT, a vaccine comprising GSC lysate, DCs, and TLR9 agonist CpG motif-containing oligodeoxynucleotides with anti-PD-L1, showed greater survival advantage and decreased the Treg cell population in the brain [98]. Combination therapy consisted of a streptavidin-granulocyte-macrophage-colony stimulating factor surface-modified bladder CSCs vaccine with anti-PD-1 showed an increase in the population of CD4^+^, CD8^+^, and CD8^+^ IFN-γ^+^ cells and a strong induction of a specific antitumor immune response against bladder cancer [99].

## 6. Conclusions

**Figure 2:**
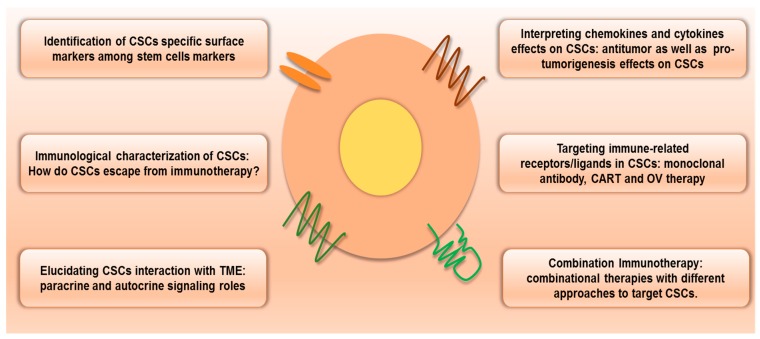
Prospective immunotherapeutic approaches to target CSCs. In order to target CSCs by immunotherapy, the identification CSCs specific surface markers and the immunological characterization of immune escaping with a complete elucidation of interaction with the TME are required. Chemokines’ and cytokines’ roles on CSCs are also related to immunotherapy. Immune receptors/ligands-based targeting by monoclonal antibody, CART and OV therapy. Owing to increase therapeutic efficacy, combination immunotherapy that consists of different approaches to target CSCs would be an ideal one (Abbreviations: CART, chimeric antigen receptor T-cells; CSCs, cancer stem cells; TME, tumor microenvironment, OV, oncolytic viruses).

As a sub population of bulk tumors, CSCs resist conventional cancer therapies, escaping from antitumor immunity through lower expression of immune recognizing receptors. The TME and niche also play vital roles in immune escaping. Various cytokines and chemokines of stromal cells and immune cells in the TME severely suppress antitumor immune activity against CSCs. Combination immunotherapies would be an ideal approach to restore antitumor immunity against CSCs. These approaches may help the complete eradication of CSCs. However, a more immunological characterization of CSCs and interactions between cellular components in the TME must be revealed. Prospective immunotherapeutic approaches to target CSCS may need to understand CSCs, their niche, and the TME together with related mechanisms (Figure 2). The TME includes various immune cells, nonimmune cells, cancer cells, and CSCs. Interaction between cellular components in the TME can affect other cells’ fates through cytokines and chemokines. These can be considered for CSC-targeted therapy. Recently, a relapse pathway of glioblastoma has been elucidated through single cell molecular analysis. Within single cells, it found three mutated genes involved in the RAS/GEF GTP-dependent signaling pathway in glioblastoma [100]. Single cell molecular analysis can be applied to reveal the interfaces of immune cells, stromal cells, cancer cells, and CSCs in the TME. This approach could elucidate the heterogeneity of tumor progression. These approaches may contribute to develop more smart CSC-targeted therapeutic approaches [101].

## Figures and Tables

**Table 1:** Cancer stem cells (CSCs) biomarkers in various cancers.

Cancer	Biomarkers	Reference
Colorectal Cancer	CD133, CD24, CD29, CD44, CD166, EpCAM, Lgr5	[41,42]
Gastric Carcinoma	CD44, CD133, CD166, EpCAM	[43]
Head and Neck Carcinomas	CD44, CD133, CD166	[44]
HCC	CD133, CD44, CD90, CD13, OVC, EpCAM	[45,46]
Prostate cancer	Integrins, CD44, CD133, CD166, Trop2, CD117, ABCG2	[47]
Ovarian cancer	CD24, CD44, CD117, CD133, ABCG2, EpCAM	[48]

Abbreviations: ABCG2, ATP-binding cassette super-family G member 2; EpCAM, Epithelial cell adhesion molecule; HCC, hepatocellular carcinoma.

**Table 2:** Immune resistance of CSCs and their mechanism in various cancers.

CSCs in Cancer	Immune Molecules	Immunological Characterization	Mechanism of Action	Reference
Glioblastoma multiforme	MHC-I, MHC-II and NKG2D	Weakly positive for MHC-I, MHC-II, and negative for NKG2D ligand molecules	Lower immunogenicity and higher suppressive activity of GBM CSCs	[56]
	MIC-1	MΦ inhibitory cytokine-1 (MIC-1)	Inhibition of MΦ/microglia phagocytosis and T-cell proliferation	[57]
	B7-H1 and soluble galectin-3	B7-H1 and soluble galectin-3	Inhibition of T-cell proliferation and induction of T regulatory cell activation	[58]
	MIF and Arg1	CSCs produce higher level of macrophage migration inhibitory factor (MIF)	CSCs released MIF induces Arg1 from MDSCs through CXCR mediated mechanism. Arg1 suppresses T-cell antitumor activity	[59]
	TLR4	Reduced TLR4 Expression	TBK1 expression through TLR4 signals to suppress RBBP5	[60]
Colon cancer	IL-4	Autocrine production of IL-4	Tumor growth and treatment resistant	[31]
	IL-4	High expression of IL-4 and expression of CRC-associated Ag COA-1	IL-4 and CIC-mediated suppression of anti-tumor T-cell responses	[61]
Melanoma	IL-2	Inhibition of IL-2 dependent T-cell action and induction of CD4^+^ CD25^+^ FoxP3^+^ regulatory T-cells	Evasion of antitumor immunity and immunotherapeutic resistance	[62]
Breast cancer	MICA, MICB and NKG2D	Downregulation of ligands, MICA and MICB for stimulatory NK cell receptor NKG2D	Autologous/allogeneic NK cells toxicity resistant	[63]
	CD47	Over expression of CD47 on CSCs by HIF-1	HIF-1-induced CD47 expression on CSCs and cancer cells escapes from phagocytosis by bone marrow-derived macrophages	[64]
	PD-L1	Enriched PD-L1 expression through EMT/βcatenin/STT3/PD-L1 signaling axis	EMT and MET upregulate PD-L1 through STT3-dependent PD-L1 N-glycosylation	[65]
Pancreatic adenocarcinoma	CXCR4	CD133^+^ and CD133^+^ CXCR4^+^ CSCs	CD133^+^ CSCs responsible for tumorigenic and highly resistant to standard chemotherapy. CD133^+^ CXCR4^+^ CSCs dictate metastatic phenotype of the individual tumor	[66]
	TGF-β1	Interaction between hCAP-18/LL-37 expression of stroma of PDAC and TGF- β1	hCAP-18/LL-37 expression of stroma of PDAC and TGF- β1 mediated tumorigenesis	[67]
Ovarian cancer	CXCR4 and CXCL12	CXCR4^+^CD133^+^ OVCAR-5 cells were resistant to cisplatin	Overexpression of ABCG2 drug transport and migrates towards CXCR4 ligand and CXCL12	[68]

Abbreviations: ABCG2, ATP-binding cassette super-family G member 2; AgCOA-1, antigen COA1; Arg1, arginase 1; B7-H1, B7 homolog 1; CICs, cancer-initiating cells; CSCs, cancer stem cells; CRC, colorectal cancer; CXCR, C-X-C chemokine receptor; CXCR4, C-X-C chemokine receptor 4; CXCL12, C-X-C motif chemokine 12; EMT, epithelial mesenchymal transition; GBM, glioblastoma multiforme; hCAP-18/LL-37, human cationic antimicrobial protein18 leucine leucine-37; HIF-1, hypoxia-inducible factor; IL-4, interleukin 4; MET, mesenchymal–epithelial transition; MHC I; major histocompatibility complex I; MHC II, major histocompatibility complex II; MICA, MHC class I polypeptide-related sequence A; MICB, MHC class I polypeptide-related sequence B; NK cells, natural killer cells; NKG2D, natural killer group 2D; PDAC, pancreatic ductal adenocarcinoma; PD-L1, programmed death-ligand 1; RBBP5, retinoblastoma binding protein; STT3,dolichyl-diphosphooligosaccharide-protein glycosyltransferase subunit STT3; TBK1, tank-binding kinase 1; TGF- β1, transforming growth factor-beta 1; TLR4, toll-like receptor 4.

**Table 3:** Recent advances in targeting CSCs by immunotherapy.

Immuno-Therapy	Targeting Approach	Cancer Model	Reference
Adoptive T-cell therapy	CAR T-cells against EpCAM antigen. Peripheral blood lymphocytes expressing EpCAM-specific chimeric antigen receptors targeted EpCAM^+^ CSCs	Prostate cancer	[72]
	CAR T-cells, targeting membrane bound IL-15	Leukemia	[73]
	CD8^+^ cytotoxic T-cells, specific for the CSCs antigen ASB4	Colon cancer	[74]
	CIK cells transduced with CAR T- cells against CD123	Acute myeloid leukemia	[75]
	Autologous CIK cells	Melanoma	[76]
	CIK cells-NKG2D ligands	HCC	[77]
	CIK cells- NKG2D ligands	Nasopharyngeal carcinoma	[78]
	NK cells from healthy donors	High-grade non-muscle invasive bladder cancer	[79]
	NK cells	Pancreatic cancer	[80]
	NK cells	Oral squamous carcinoma	[81]
	γδ and CD8^+^ T-cells	Breast cancer	[82]
DC-based vaccine	CSCs lysate-pulsed DCs	Malignant melanoma	[83]
	CSCs lysate-pulsed DCs	Squamous cell carcinoma	[84]
	DCs charged with total lysates of Panc-1 CSCs	Pancreatic cancer	[85]
	DCs loaded with NANOG peptide	Ovarian cancer	[86]
	ALDH^high^ SCC7 specific CSCs-DCs	Squamous cell cancer	[87]
	ALDH^high^ D5 CSCs-DCs	Metastatic melanoma	
	ALDH^high^ CSC-pulsed DCs	Metastatic melanoma	[88]
	ALDH^high^ CSC-pulsed DCs	Squamous cell cancer	
Oncolytic virotherapy	Oncolytic herpes simplex virus armed with IL-12	Glioblastoma	[89]
	Oncolytic adenovirus targeting CD133^+^ CSCs	Glioblastoma	[90]
	Oncolytic vaccinia virus (GLV-1h68) targeting ALDH^high^ stem cell-like cancer cells	Breast cancer	[91]
	Oncolytic vaccinia virus targeting ID8-T tumor model that harbors CD44^+^ CD117^+^ cancer-initiating cells	Ovarian cancer	[92]
	Cancer-favoring oncolytic vaccinia virus: stem-cell-like colon (CD133^+^ and CD44^+^) cancer cells	Colon cancer	[42]
	Oncolytic measles viruses: targeting CD133^+^ tumor-initiating cells	HCC	[93]
	Cancer-favoring oncolytic vaccinia virus: metastatic hepatocellular carcinoma (CD44^+^)	HCC	[46]
Others	Monoclonal antibody against Lgr5	Colon cancer	[94]
	IFN-β therapy: targeting type I IFN signaling	Triple negative breast cancer	[49]
	Blockade of the IL-8 receptor	Breast cancer	[95]
Combination therapy	DC-based vaccine in combination with anti-PD-L1 and anti-CTLA-4	Melanoma	[96]
	Oncolytic herpes simplex virus in combination with anti-PD-1 and anti-CTLA-4	Glioblastoma	[97]
	STDENVANT (a vaccine comprising of GSC lysate, DCs, and TLR9 agonist CpG motif-containing oligo-deoxynucleotides) in combination with anti-PD-L1	Glioblastoma	[98]
	CSCs vaccine (streptavidin-granulocyte-macrophage-colony stimulating factor surface-modified bladder CSCs) in combination with anti-PD-1	Bladder cancer	[99]

Abbreviations: ALDH, aldehyde dehydrogenase; CAR, chimeric antigen receptor; CCR7, CC-chemokine receptor 7; CIK cells, Cytokine-induced killer cells; CRC, colorectal cancer; CSCs, cancer stem cells; CTLA-4, cytotoxic T-lymphocyte–associated antigen 4; CXCR1, C-X-C chemokine receptor 1; DC, dendritic cells; HCC, hepatocellular carcinoma; IFN-β, interferon-beta; IFN-γ, interferon-gamma; IL-8, interleukin 8; IL-12, interleukin 12; NK, natural killer; NKG2D, natural killer group 2D; PD-1, Programmed cell death-1; PD-L1, programmed death-ligand 1; TLR9, toll-like receptor 9.
